# Feline Parvovirus Seroprevalence Is High in Domestic Cats from Disease Outbreak and Non-Outbreak Regions in Australia

**DOI:** 10.3390/v12030320

**Published:** 2020-03-16

**Authors:** Elizabeth Jenkins, Conor Davis, Maura Carrai, Michael P. Ward, Susan O’Keeffe, Martine van Boeijen, Louise Beveridge, Costantina Desario, Canio Buonavoglia, Julia A. Beatty, Nicola Decaro, Vanessa R. Barrs

**Affiliations:** 1 Sydney School of Veterinary Science, Faculty of Science, University of Sydney, Camperdown 2050, Australia; elizabeth.jenkins@sydney.edu.au (E.J.); cdav6397@uni.sydney.edu.au (C.D.); maura.carrai@sydney.edu.au (M.C.); michael.ward@sydney.edu.au (M.P.W.); julia.beatty@sydney.edu.au (J.A.B.); 2 School of Veterinary and Life Sciences, Murdoch University, Murdoch 6150, Australia; 3 Perth Cat Hospital, West Leederville 6007, Australia; drmartine@perthcat.vet; 4 Bedford-Dianella Vet Centre, Bedford 6052, Australia; louise_bedfordvet@hotmail.com; 5 Department of Veterinary Medicine, University of Bari, Valenzano, 70121 Bari, Italy; costantina.desario@uniba.it (C.D.); canio.buonavoglia@uniba.it (C.B.); nicola.decaro@uniba.it (N.D.)

**Keywords:** *Carnivore protoparvovirus*, feline parvovirus, feline panleukopenia, haemagglutination inhibition, seroprevalence

## Abstract

Multiple, epizootic outbreaks of feline panleukopenia (FPL) caused by feline parvovirus (FPV) occurred in eastern Australia between 2014 and 2018. Most affected cats were unvaccinated. We hypothesised that low population immunity was a major driver of re-emergent FPL. The aim of this study was to (i) determine the prevalence and predictors of seroprotective titres to FPV among shelter-housed and owned cats, and (ii) compare the prevalence of seroprotection between a region affected and unaffected by FPL outbreaks. FPV antibodies were detected by haemagglutination inhibition assay on sera from 523 cats and titres ≥1:40 were considered protective. Socioeconomic indices based on postcode and census data were included in the risk factor analysis. The prevalence of protective FPV antibody titres was high overall (94.3%), even though only 42% of cats were known to be vaccinated, and was not significantly different between outbreak and non-outbreak regions. On multivariable logistic regression analysis vaccinated cats were 29.94 times more likely to have protective FPV titres than cats not known to be vaccinated. Cats from postcodes of relatively less socioeconomic disadvantage were 5.93 times more likely to have protective FPV titres. The predictors identified for FPV seroprotective titres indicate targeted vaccination strategies in regions of socioeconomic disadvantage would be beneficial to increase population immunity. The critical level of vaccine coverage required to halt FPV transmission and prevent FPL outbreaks should be determined.

## 1. Introduction

Feline panleukopenia (FPL), caused by *Carnivore protoparvovirus* 1 (Order *Ortervirales*, Family *Parvoviridae*, Subfamily *Parvovirinae*), is characterised by severe enteritis and immunosuppression and has high morbidity and mortality. Feline parvovirus (FPV) causes 95% of cases, with the remaining 5% caused by canine parvovirus (CPV), including the antigenic variants CPV-2a, -2b, and 2c, also known as “CPV-2a-like” viruses [1]. 

In Australia FPL has been encountered only sporadically since the mid-1970s when the use of effective vaccines became widespread. Between 2014 and 2018, FPL re-emerged in eastern Australia, causing multiple epizootic outbreaks with high mortality predominantly among shelter-housed cats [2]. FPV was identified as the causative agent of the first outbreak in 2014 in the state of Victoria [2]. Outbreaks in New South Wales (NSW) first occurred in 2016 and were caused by a different and distinctive FPV strain, ruling out geographic spread from Victoria. Most affected cats were unvaccinated, or vaccination history was unknown; hence low population immunity was hypothesised to be a major driver of FPL re-emergence [1,2].

Serum antibody titres to FPV, determined using virus neutralisation (VN) or haemagglutination inhibition (HI) assays or ELISAs (laboratory-based or point-of-care), are used to predict protection from disease [3,4,5,6]. A cut-off ≥1:40 is used widely to define the minimum HI titre that confers serological protection, but a range of cut-offs from 0 to ≥1:80 have been reported [3,4,7,8,9].

A single previous study of the seroprevalence of FPV antibodies in Australian cats (*Felis catus*) undertaken in 1981 and involving 92 unowned, free-living cats in south-eastern Australia, found a seroprevalence of 79%, suggesting widespread exposure to FPV [10]. FPV seroprevalence data among shelter-housed cats or owned cats in Australia have not been reported previously. Factors associated with FPV seropositivity in other regions include previous vaccination [11], increasing age, being owned, and being desexed [8,9,12,13].

The aim of this study was to determine (i) FPV seroprevalence and predictors of seropositivity among shelter-housed and owned cats, and (ii) whether seroprevalence differed in these two cohorts between an outbreak location in eastern Australia (Sydney) and a region with no recent history of FPL outbreaks in western Australia (Perth).

## 2. Materials and Methods

### 2.1. Study Populations

Blood samples were collected from four groups of cats: (i) owned and (ii) shelter-housed cats from Sydney, NSW, a region in which recurrent FPL season outbreaks have been occurring since December 2016 [2]; and (iii) owned and (iv) shelter-housed cats from Perth, Western Australia (WA), where no FPL outbreaks have been reported in recent decades. Blood samples were collected from a single large shelter in Perth and from three smaller shelters in Sydney. Blood samples were collected from owned cats presenting for veterinary consultations collected from seven veterinary hospitals in Sydney and from two veterinary hospitals in Perth. In Sydney, four of the veterinary hospitals included were in close proximity to the previously reported outbreaks and were participating in a subsidised vaccination and desexing scheme offered by a charitable feline organisation. A power analysis was performed to determine the number of cats to be sampled. Based on an estimated minimum seroprevalence of 15% to 40% among shelter-housed cats, and 50% to 70% among owned cats, the minimum total sample size required to detect a difference in seroprevalence between these groups, with confidence set at 95%, significance of *p* < 0.05, and power ≥80%, was 200 cats overall [14]. The sample size required for risk factor analysis an odds ratio of 1.5 to be detected with 95% confidence and 40% precision assuming a 20% prevalence of the risk factor in cats with non-protective antibody titres, and a minimum ratio of cats with protective titres to cats with non-protective titres of 1.5 was 142 cats with non-protective titres [15], which would be achieved by sampling 400 cats overall (100 in each group).

### 2.2. Sampling and Data Collection

Whole blood (1–3 mL) was collected via jugular venepuncture and serum was separated and stored frozen at −20 °C for up to 4 weeks, then transferred for storage at −80 °C until batch testing. Prospective sampling was performed from June 2018 to July 2019. Additionally, 35 stored sera collected from July to August 2015, from owned cats in Perth (non-outbreak region) were included.

Data collected for each cat sampled included age, sex, desexing status, breed, location (post code), reason for presentation (health check, injured or unwell, vaccination, desexing, stray, other), time in possession of owner or shelter, other dogs or cats in the household (yes or no), health status (healthy, defined as fit to vaccinate, sick or injured), current medications, feline leukemia virus (FeLV) antigen status, feline immunodeficiency virus (FIV) antibody status, FPV vaccination status, time since last vaccination (<6 months ago, 6–12 months ago, 1–3 years ago, >3 years ago and type of vaccination (attenuated or inactivated). The study was approved by the University of Sydney Animal Care and Ethics Committee, Approval No. 2017/1218.

### 2.3. Haemagglutination Inhibition (HI) Serology

FPV antibody titres were determined by HI assays using sera after complement inactivation, as previously described, with minor modifications [16,17]. Briefly, serum was diluted in phosphate-buffered saline (PBS) pH 5.6 and mixed with an equal amount v/v of undiluted porcine red blood cells (RBC), then centrifuged after overnight incubation at 4 °C, at 800× *g* for 5 min at 4 °C and the supernatant was harvested. HI assays were performed in 96-well V-bottom plates (Nuova Aptaca, Canelli-AT, Italy) using 25 µL of diluted sera mixed with 25 µL of an FPV suspension containing 10 haemagglutinating units (FPV field strain 20/05), and doubling dilutions from 1:10 to 1:2560. Plates were incubated at room temperature for 1 h, then at 4 °C for 30 min, after which 50 µL of a solution containing 0.1% porcine RBC suspension and 2% foetal bovine serum (Mediatech, Inc., Manassas, VA, USA) in PBS pH 7.2 were added to each well. Plates were read after overnight incubation at 4 °C. A titre of ≥1:40 was considered protective. 

### 2.4. Statistical Analysis

Descriptive analysis was performed by creating frequency distributions for categorical variables and estimating medians for continuous variables. Statistical analyses were performed using the software package IBM SPSS Statistics v24. For univariable analysis of the association between FPV protection status and categorical predictor variables, Chi-squared tests of association were used, with a *p*-value set at 0.05 for statistical significance and a protective titre was defined as an HI titre ≥1:40 [4,18]. Seroprevalence confidence intervals (95%) were calculated using the Clopper–Pearson exact test. 

Socioeconomic data were sourced from the Australian Bureau of Statistics Socio-Economic Indexes for Areas (SEIFA) data cube from 2011 based on postcode and published by the Australian Bureau of Statistics in 2013, and the index of relative socio-economic disadvantage (IRSD), the index of relative socio-economic advantage and disadvantage (IRSAD), the index of economic resources (IER), and the index of education and occupation (IEO) [19] were extracted. Index values for each cat included in the study were based on its reported postcode. The association between each of the four indices and FPV protection status (protective vs. non-protective titre) was assessed using Mann–Whitney U tests, with a *p*-value of 0.05 used for statistical significance.

A multivariable logistic regression model was fit to the data using predictors significantly (*p* < 0.05) associated with FPV protection status analysis. A separate logistic regression analysis was performed using SEFIA variables included as categorical variables (above median vs. median or below median). The association between FPV titre and vaccination status was assessed with Kruskal–Wallis one-way analysis of variance, with a *p*-value of 0.05 used for statistical significance.

## 3. Results

### 3.1. Animals

**Figure 1:**
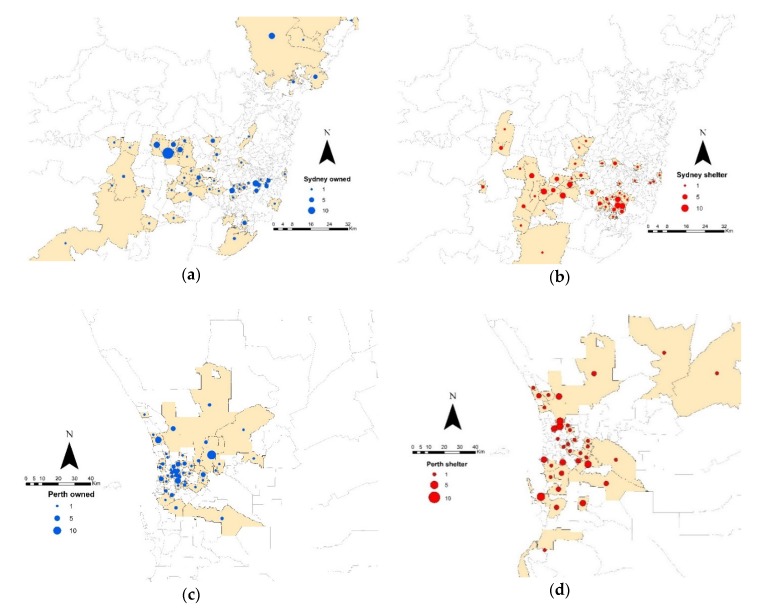
Geographic origin of samples collected in this study. Samples collected from shelter-housed cats are represented by blue dots, and samples from owned cats are represented by red dots. The size of the dots is proportional to the number of samples collected from one postcode area. The postcode areas sampled are shaded in beige. Owned (**a**) and shelter-housed (**b**) cats in the outbreak region (Greater Sydney); owned (**c**) and shelter-housed (**d**) cats in the non-outbreak region (Greater Perth).

A total of 523 cats were included in the study comprising 282 cats from Sydney (outbreak-region) and 241 cats from Perth (non-outbreak region). The geographic origins of cats sampled in the study are shown in Figure 1. Of the 523 cats, 234 were shelter-housed and 289 were owned. The median age of cats overall was 1.75 years (range: 7 weeks to 21 years). The median age of cats within each group was 1 year and 1.1 years for Sydney owned and shelter-housed cats, respectively and 10 years and 1 year for Perth owned and shelter-housed cats, respectively. The median age of cats overall from Sydney and Perth was 1 year and 3.5 years, respectively.

Of cats from Sydney, 29% of owned and 47% of shelter-housed cats were vaccinated; of cats from Perth, 19% and 78% of owned and shelter-housed cats, respectively were vaccinated. Overall, 37.5% and 48.1% of cats were vaccinated from Sydney and Perth, respectively. Other categorical data are shown in Table 1.

### 3.2. Seroprevalence and Variables Associated with a Protective FPV Titre

**Figure 2:**
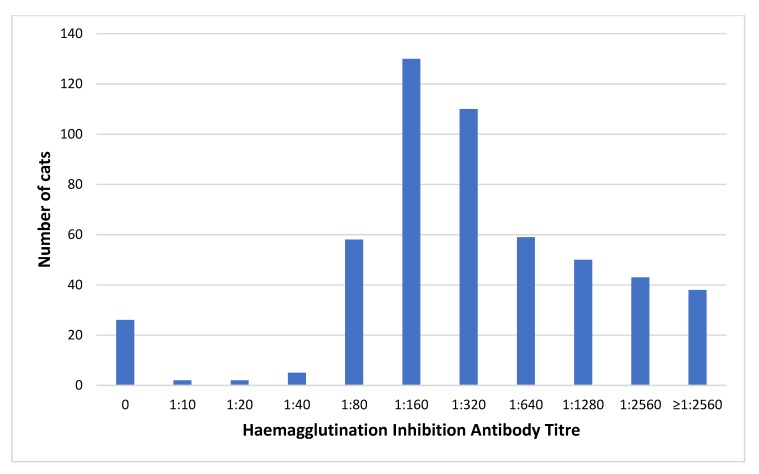
Frequency distribution of serum FPV haemagglutination inhibition titres among all cats tested in this study (*n* = 523).

Overall, 493 of the 523 cats (94.3%) had protective FPV antibody titres. The frequency distribution of FPV HI titre results is shown in Figure 2. There was a significant difference in the magnitude of FPV titres between vaccinated cats and cats that were unvaccinated or of unknown vaccination status (*p* < 0.001). Among seropositive cats, the median HI titre was 1:160 for unvaccinated/unknown vaccination status cats and 1:640 for vaccinated cats (*p* < 0.001).

For the univariable analysis, cats of unknown vaccination status (*n* = 200) and unvaccinated cats (*n* = 105) were considered as one group. There was no significant difference in the presence of FPV protective titres between cats of unknown vaccination status (91%; CI 86.15%–94.58%) and unvaccinated cats (89.5%; CI 82.01%–94.65%; Χ^2^ = 0.1744, *p* = 0.6762). In the univariable analysis, factors significantly associated with a protective FPV titre were study group, vaccination status (Table 1), and three of the four SEIFAs (IRSD *p* = 0.001; IRSAD *p* = 0.001; IEO *p* = 0.002). 

A multivariable logistic regression analysis was performed to predict FPV protection status. Using a stepwise logistic regression model fit to the significant variables in the univariable analysis, group, and vaccination status, a large standard error for the Sydney shelter group indicated that the variable “group” was problematic to include in this model. A more stable model was achieved using the variables of ownership (owned, shelter-housed) and vaccination status (vaccinated vs. unvaccinated/unowned). The model adequately fit the data (Hosmer–Lemeshow, *p* = 0.077 and Nagelkerke r^2^ value = 0.164). In this model the predictors of having a protective titre were being vaccinated (odds ratio (OR) = 29.94) and being a shelter-housed cat (OR = 2.69) (Table 2).

For logistic regression analysis of SEIFA data, when re-categorised as above median vs. median or below median, the IRSD was the best predictor of protective status and cats from postcodes with above median index of disadvantage (relatively less disadvantaged) were 5.93 times more likely to have protective FPV titres (Table 3). The model adequately fit the data (Hosmer–Lemeshow, *p* = 0.999 and Nagelkerke r^2^ value = 0.080)

## 4. Discussion

The prevalence of protective FPV antibody titres was surprisingly high, exceeding 94% in cats studied. Among vaccinated cats the high prevalence of seroprotection (99.5%) was not unexpected, since FPV vaccines are highly effective in inducing long-lasting humoral immunity [4]. A similarly high FPV seroprevalence among vaccinated cats (97%–98%) was reported in North America in 2004, and in Austria in 2016 [20] (Table 4). However, high seroprevalence among vaccinated cat populations is not universal. For example, an investigation of 350 cats in Germany found that 23% of cats that had received FPV vaccinations in accordance with current global vaccination guidelines had no detectable serum antibodies [8,11]. In that study, in addition to vaccination status, risk factors for being seronegative were comorbid disease and glucocorticoid administration.

We identified that a history of FPV vaccination was a strong predictor of protective FPV antibody titres, and the OR of 29.9 among vaccinated cats, was similar to that found in the German study, in which the OR for vaccinated cats having protective FPV titres was 24.8 [8]. 

The high proportion of seroprotective titres among cats that were unvaccinated or of unknown vaccination status was unexpected. Although it is likely that some cats of unknown vaccination status were in fact vaccinated, there was no significant difference in seroprotection prevalence between this group and unvaccinated cats. Serosurveillance of unvaccinated domestic cats in other countries revealed an FPV seroprevalence ranging from 8% to 96%, although <50% of cats were seropositive in 7 of 10 studies [9,12,13,21,22,23,24,25,26,27] (Table 4). We used a cut-off HI titre of 1:40 to define seroprotection to allow comparison with the majority of published studies. The WSAVA vaccination guidelines panel considers that the presence of a serum antibody, regardless of titre, is protective [11]. Using that approach would have made little difference to our results, since only five cats had HI titres above zero but less than 1:40.

Our results indicate that cats in Australia are commonly exposed to *Carnivore protoparvovirus 1* in the field. Unvaccinated cats could have had environmental exposure to field or vaccine strains of FPV or CPV. Cats are primarily exposed to FPV through contact with fomites, since this small non-enveloped virus is extremely environmentally resilient and capable of persisting for >12 months in favourable conditions [30,31]. Environmental contamination is largely due to faecal shedding of the virus by infected cats, who shed the virus at very high titres for several weeks post-infection. Attenuated vaccine virus strains are also shed in the faeces of vaccinated cats subsequent to replication in the gut [2,32]. 

The extent to which exposure to CPV in cats provides protection against FPV infection has not been investigated. CPV is antigenically similar to FPV, since these viruses only differ by several amino acid residues in the viral capsid [33]. In experimental studies where cats were inoculated with CPV-2c or CPV-2a orally, all cats developed FPV neutralising (VN) antibody titres, but of a lower magnitude than VN titres to CPV, suggesting that immunity to FPV induced by CPV would be shorter in duration than that induced by FPV [34]. Similarly, sera from cats immunised with attenuated FPV vaccines neutralise CPV, although neutralising antibody titres are two- to eight-fold lower than those induced by CPV, consistent with a shorter duration of immunity [5,35]. Interestingly, seropositive cats that were unvaccinated or of unknown vaccination status in our study had three-fold lower median HI titres than vaccinated cats. This could be due to a number of factors, including exposure to CPV but not FPV, or a decline in FPV titre over time after initial exposure in unvaccinated cats in contrast to vaccinated cats, which may have been re-vaccinated repeatedly over time. In cats that develop FPL, exposure to field strains of parvoviruses by natural infection often induces higher VN or HI titres than those induced after vaccination with a homologous strain [34,36]. Whether this is true for cats subclinically infected with FPV is not known. We did not find an association between the presence of dogs in the household and FPV seropositivity, although this is not surprising since exposure to parvoviruses is often indirect. Future investigations to determine CPV- and FPV-specific antibody titres simultaneously are warranted in order to better understand the dynamics of the role of exposure to heterologous parvoviruses in FPV immunity among unvaccinated cats. In addition, or alternatively, the parvoviral strains that cats have been exposed to can be determined by molecular sequencing of persistent episomal parvoviral DNA in blood monocytes post-infection. In two of three studies that used this technique in healthy seropositive cats from Italy and Vietnam, exposure to CPV was found to be as, or more, frequent than to FPV [37,38,39]. 

The high rate of FPV seropositivity among cats not known to be vaccinated also indicates that most infections were likely subclinical or caused only mild disease. The development and severity of FPL in naïve cats is dependent on the interplay of multiple host factors (e.g., age, immune status) and viral factors (e.g., inoculating dose), as well as the presence, in some cases, of co-pathogens including intestinal parasites and other enteric viruses (e.g., bocaviruses) [40,41]. Since FPV is profoundly immunosuppressive in cats, opportunistic pathogens, such as bacteria and fungi, can also contribute to the development of severe clinical disease [42,43]. The role of co-pathogens, if any, in the Australian FPL outbreaks, has not been investigated.

Contrary to our expectations, we did not find a significant difference in FPV seroprevalence between outbreak and non-outbreak regions. The high rate of immunity in the outbreak population tested may reflect “boosting” of population immunity among unvaccinated cats due to widespread exposure to FPV in the field, since sera were collected one to two years after the first epizootic outbreaks occurred in Sydney. Supporting this, the seroprevalence in the outbreak population was high despite the overall proportion of cats known to be vaccinated (37.5%) being lower than that of the non-outbreak population (48%). 

Unlike several other studies in which FPV seropositivity was associated with age >6 months or age >1 year, reflecting an increasing likelihood of exposure over time, and especially within the first year of life, we did not find an association with age [9,12,13]. 

The significant difference in seroprevalence among the four groups of cats detected in univariable analysis was likely influenced by the different vaccination proportions among these groups. Owned cats in Sydney were younger than those in Perth because we targeted veterinary clinics in close proximity to the previous FPL outbreaks that were enrolling cats for subsidised desexing programs in the outbreak region.

We could not include group in the multivariable analysis together with vaccination status because of the large standard error (SE) associated with the Sydney shelter group, likely a consequence of the uniform presence of protective titres in this study group. Another potential limitation of our study was that the number of seronegative results was smaller than expected, which might have limited our ability to identify significant predictors of seropositivity in logistic regression analyses. The finding that shelter-housed cats were 2.65 times more likely to have protective FPV titres than owned cats may have been influenced by differences in their exposure history.

Our finding that cats from postcodes of relatively less disadvantage were 5.93 times more likely to have protective FPV titres is useful to inform strategies to achieve more homogeneous immunity to FPV in the cat population, such as the locations and frequency of community companion animal vaccination and pet health educational events provided by veterinary professional organisations and animal charities. The IRSD used in this study is based on measures of wealth and income, including household income, employment status, and level of education obtained from census data [19]. 

Based on FPV seroprevalences of 68.5% to 70.6% among owned populations of cats, it has been suggested that epizootic outbreaks will not occur when the seroprevalence is >70% [11,40]. However, the critical level of vaccine coverage required to prevent FPL outbreaks is unknown, since the basic reproduction number (*R*_0_) of FPV (i.e., the number of new cases of infection generated by the first infectious individual in a completely susceptible population), has not been modelled for *Carnivore protoparvovirus 1* from outbreak data [44]. Simplistically, the critical level of vaccine coverage, or the fraction of the population that is required to be immunised to halt transmission of a pathogen within a population, can be calculated as 1 − 1/*R*_0_ [44]. For pathogens with a high *R*_0_, the critical level of vaccine coverage is very high and outbreaks can occur with relatively small fluctuations in population immunity, even though the vast majority of the population is immune [45]. Disease outbreaks can occur when inhomogeneous vaccine coverage results in susceptible pockets of the population occurring in an otherwise protected population. 

While vaccination is undoubtedly the most effective strategy to protect a cat against FPL, administration of FPV vaccines to seropositive cats is unnecessary, ineffective in boosting immunity, and has the potential for adverse effects [46]. FPV antibody titre testing is being increasingly used by veterinarians to determine the timing of vaccine administration in owned cats. Point-of-care (POC) tests with high positive predictive values (PPV) are desirable and enable the decision to vaccinate to be made during the same consultation as testing. POC tests with high PPV are available for FPV titre testing in cats, and since PPV increases with seroprevalence, use of these tests among similar populations of Australian cats to those tested here, would be unlikely to result in seronegative animals not being vaccinated, since the likelihood of incorrect identification of a cat as being seropositive would be very low [6]. 

## Figures and Tables

**Table 1:** Descriptive and univariable analysis of risk factors potentially associated with a protective feline parvovirus serum haemagglutination antibody titre (≥1:40) for 523 shelter-housed and owned cats tested from Sydney and Perth.

Variable	Category	Total No.	Protective FPV Titre		95% CI	Χ^2^-Value	*p*-Value ^1^
			No.	%			
Group	Perth owned	117	112	95.7	90.4–96.8	11.733	**0.008**
	Perth shelter	124	115	92.7	86.7–96.6		
	Sydney owned	172	156	90.7	90.9–96.7		
	Sydney shelter	110	110	100	96.7–100		
Shelter vs. Owned	Shelter	234	225	96.	92.8–98.2	2.798	0.094
	Owned	289	268	92.7	89.1–95.5		
Location	Perth	241	227	94.2	90.4–96.8	0.004	0.947
	Sydney	282	266	94.3	90.9–96.7		
Age	<1 year	205	188	91.7	87.05–95.1	4.154	0.125
	1–8 years	232	222	95.7	92.2–97.9		
	>8 years	86	83	96.5	90.1–99.3		
Sex	Male	241	224	93	89–95.8	2.202	0.138
	Female	250	240	96	92.8–98.1		
Desexing Status	Desexed	275	263	95.6	92.5–97.7	0.755	0.686
	Intact	195	183	93.9	89.5–96.8		
	Unknown	19	18	94.7	74.0–99.9		
Breed	Domestic	418	403	96.4	94.2–98.0	0.087	0.087
	Non-domestic	53	50	94.3	84.4–98.9		
Outdoor Access	Indoors only	99	91	91.9	84.7–96.5	0.788	0.375
	Outdoor access	253	239	94.5	90.9–96.9		
Dogs in the House	No	97	96	99.0	94.4–100	0.519	0.471
	Yes	50	50	100	92.9–100		
Source	Breeder/Pet shop	51	48	94.1	83.8–98.8	4.435	0.109
	Shelter/Stray	275	259	94.2	90.7–96.6		
	Other	108	107	99.1	95.0–100		
Health Status	Healthy	417	396	95.0	92.4–96.9	0.085	0.770
	Sick/Injured	71	68	95.8	88.1–99.1		
Vaccination Status	Vaccinated	218	217	99.5	97.5–100	17.617	**<0.001**
	Unvaccinated or Unknown	305	276	90.5	86.6–93.5		
Vaccination Type	MLV only	114	113	99.1	95.2–100	0.336	0.846
	Inactivated only	18	18	100	81.5–100		
	Both	20	20	100	83.2–100		
Time Since Last Vaccination	<6 months	22	21	95.5	77.16–99.9	1.651	0.199
	6–12 months	126	126	100	97.1–100		
	1–3 years	38	38	100	90.8–100		
	>3 years	22	22	100	84.6–100		
	Unknown	10	10	100			
Medications	No	446	419	94.0	91.3–96.0	0.565	0.452
	Yes	77	74	96.1	89.0–99.2		
FeLV Antigen Status	Negative	82	82	100	95.6–100	n/a	n/a
	Positive	0	0				
FIV Antibody Status	Negative	105	104	99.1	94.8–100	0.23	0.631
	Positive	24	24	100	85.8–100		

^1^ Significant *p*-values (<0.05) are in bold text; FPV: feline parvovirus; FeLV: feline leukemia virus; FIV: feline immunodeficiency virus; MLV: modified live virus.

**Table 2:** Results of logistic regression analysis using model of best fit containing the variables: vaccinated, Sydney (outbreak) location, and shelter-housed.

	B	SE	Wald	df	*p*-Value	OR	95% CI
Vaccinated	3.293	1.024	10.346	1	0.001	29.94	3.62–200.4
Shelter-Housed	0.988	0.416	5.635	1	0.018	2.685	1.19–6.07
Constant	1.140	0.407	7.843	1	<0.001	3.128	–

B: beta coefficient; SE: standard error; df: degrees of freedom; OR: odds ratio; CI: confidence interval.

**Table 3:** Results of logistic regression analysis using model of best fit to analyse the index of relative socio-economic disadvantage (IRSD).

	B	SE	Wald	df	*p*-Value	OR	95% CI
IRSD	1.780	0.629	7.995	1	0.005	5.928	1.73–20.35
Constant	2.369	0.240	97.486	1	0.000	10.684	–

B: beta coefficient; SE: standard error; df: degrees of freedom; OR: odds ratio; CI: confidence interval; IRSD: index of relative social disadvantage.

**Table 4:** Seroprevalence of protective serum antibody titres against FPV in domestic cats from different geographic regions.

Year of Sampling	Country	No. Cats	Origin of Cats	Proportion Known to Be Vaccinated (%)	FPV Seroprevalence (%)	Reference
1981	Australia	92	stray/feral	0	79	[10]
1989	UK	45	free-ranging farm cats	0	96	[20]
1997	Vietnam (North)	69	unowned	0	54	[21]
1998	Vietnam (South)	50	unowned	0	44	[22]
1998–2000	Saudi Arabia	13	feral	0	8	[23]
2004	Ecuador	52	owned/feral	0	67	[24]
2005	USA (Florida)	61	feral	0	33	[25]
2007	France	469	owned/stray	0	25	[12]
2010	USA (Florida)	347	shelter	0	40	[9]
2013	Russia	60	owned	0	45	[26]
2017–2018	Italy	151	stray	0	46	[13]
1998–2001	Costa Rica	97	owned	17	93	[27]
2001	USA (Colorado)	276	owned	U	69	[3]
2001	Guatemala	30	owned	27	50	[28]
2003	USA and Canada	272	owned	100	98	[7]
2011–2012	Germany	350	owned	81	71	[8]
2012–2014	Germany	112	owned	64	64	[29]
2016	Austria	92	owned	100	97	[19]

U: unknown.
